# From continuous signals to accountable care: a trajectory-based framework for wearable monitoring in older adults

**DOI:** 10.3389/fdgth.2026.1998814

**Published:** 2026-10-28

**Authors:** Jose E. Krieger

**Affiliations:** Instituto do Coração (InCor), Hospital das Clínicas HCFMUSP, Faculdade de Medicina, Universidade de São Paulo, São Paulo, Brazil

**Keywords:** cardiovascular health, digital biomarkers, digital health, frailty, older adults, remote patient monitoring, wearable technologies

## Abstract

Wearable technologies can continuously measure cardiovascular, physiological, and behavioral signals outside clinical settings, but more measurement does not necessarily produce better care. This limitation is particularly important in older adults, in whom frailty, multimorbidity, polypharmacy, cognitive impairment, altered vascular physiology, and caregiver dependence modify both signal reliability and clinical meaning. We argue that wearable monitoring in later life should move from population thresholds toward individualized physiological trajectories and from automated alerts toward accountable clinical action. We propose a trajectory-to-action framework based on five elements: individual baseline, within-person deviation, multimodal concordance, geriatric contextualization, and predefined clinical response. The framework is conceptual and has not been prospectively validated; its clinical utility, safety, implementation feasibility, and impact on outcomes require prospective testing. In this model, an alert is not a diagnosis but a request for attention whose recipient, response time, confirmatory pathway, and escalation rules are established in advance. Evaluation should therefore extend beyond sensor accuracy to include actionability, alert burden, clinical utility, equity, and net benefit. We propose that wearable monitoring in older adults should be evaluated by whether continuous signals can be translated into proportionate, context-aware, and accountable care.

## Introduction: the problem is no longer measurement

Wearable devices have moved cardiovascular monitoring beyond the clinic. Heart rate, rhythm, activity, sleep, oxygen saturation, temperature, blood pressure-related signals, and other physiological measures can increasingly be captured during everyday life. Recent reviews have described the expanding technological landscape and its applications across cardiovascular prevention, arrhythmia detection, heart failure, rehabilitation, and chronic disease management (1). Yet the principal translational problem is no longer whether continuous data can be collected. It is whether those data can be converted into information that changes care.

This problem is especially important in older adults. Multimorbidity, frailty, polypharmacy, arrhythmia, vascular stiffness, low peripheral perfusion, cognitive impairment, sensory limitations, and dependence on caregivers may alter device performance, adherence, interpretation, and the consequences of an alert (2, 3). At the same time, deterioration in later life often develops gradually rather than as an abrupt crossing of a diagnostic threshold. Reduced walking, fragmented sleep, increasing resting heart rate, altered blood pressure, declining oxygen saturation, weight change, or loss of regular daily activity may each be nonspecific. Their joint deviation from an individual's previous state, however, may signal loss of physiological stability.

Digital biomarkers of frailty and mobility are increasingly measurable in real-world settings. Wearable-derived activity patterns and digital biomarkers have been shown to characterize and predict physical functioning in older adults, while broader studies suggest potential for detecting frailty outside conventional clinical assessments (4, 5). Nevertheless, evidence remains heterogeneous, and much of the literature has focused on classification rather than longitudinal clinical use. Recent international consensus work has accordingly identified longitudinal measurement, contextual information, algorithm validation, and integration with healthcare systems as priorities for wearable research in aging populations (6).

We propose that the next step should be more fundamental: the unit of clinical interpretation should shift from isolated measurements and population thresholds to individualized, multimodal trajectories linked to explicit responsibility for action (Figure 1). The trajectory-to-action framework presented here is a conceptual, non-validated model rather than an established clinical pathway. Its clinical utility, safety, implementation feasibility, and impact on patient-centered outcomes require prospective validation and implementation testing in defined use cases. This Perspective is not a systematic review; the literature was selected narratively to represent key evidence and recent work most directly relevant to wearable sensing, frailty and mobility, remote monitoring, blood-pressure measurement, implementation, and clinical governance.

**Figure 1:**
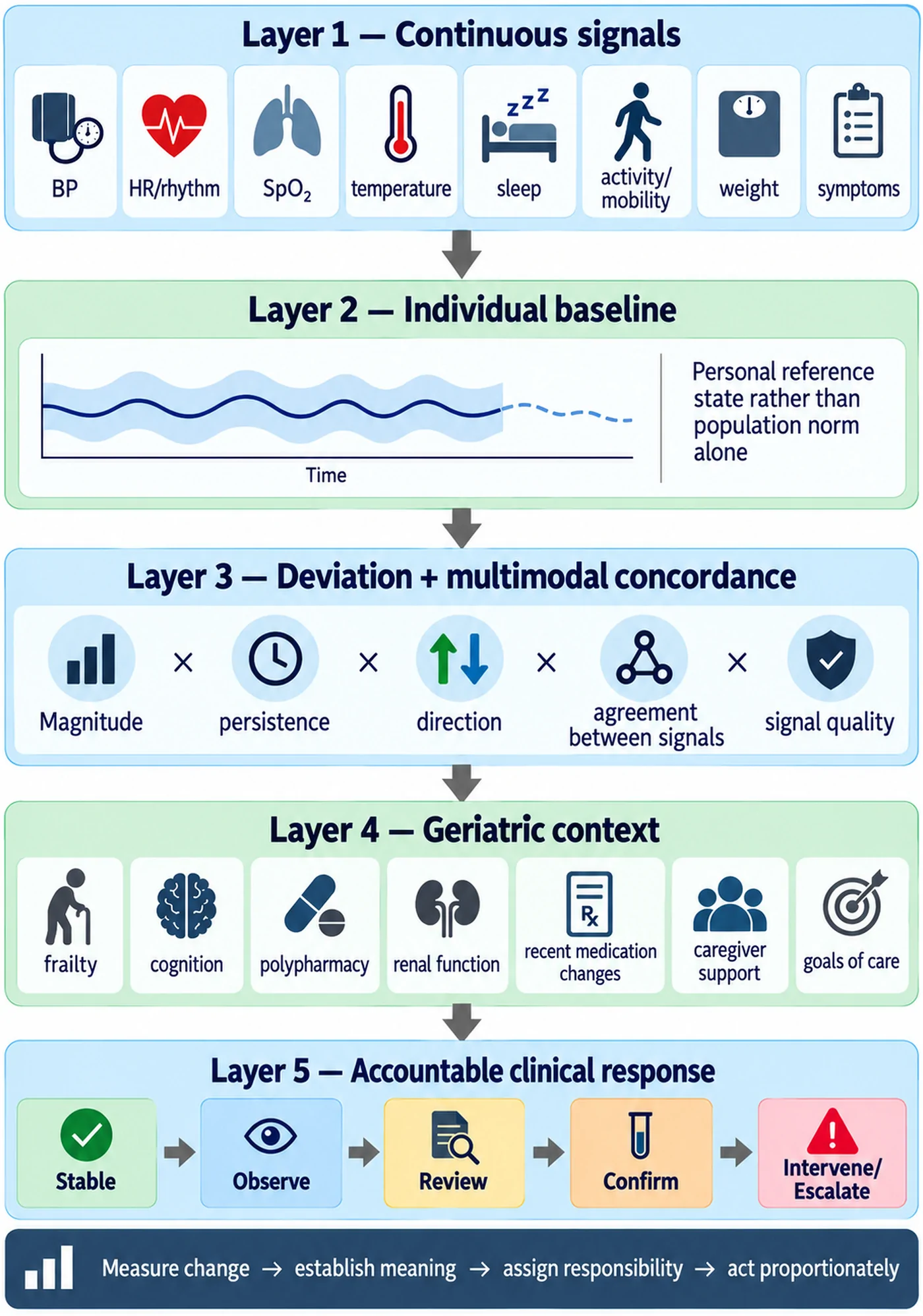
From continuous signals to accountable care: the trajectory-to-action framework. Wearable and connected-device signals, including blood pressure (BP), heart rate and rhythm (HR/rhythm), oxygen saturation (SpO₂), temperature, sleep, activity or mobility, weight, and symptoms, are first interpreted relative to an individual baseline rather than population thresholds alone. Subsequent changes are evaluated according to their magnitude, persistence, direction, concordance across modalities, and signal quality. Their clinical significance is then modified by geriatric context, including frailty, cognition, polypharmacy, renal function, recent medication changes, caregiver support, and goals of care. This integrated assessment is proposed to inform a graded response ranging from stability and observation to clinical review, confirmation, intervention, or escalation. The framework emphasizes a trajectory-based model in which continuous monitoring is useful only when physiological change is translated into meaning, linked to defined responsibility, and followed by proportionate clinical action. This is a conceptual, non-validated model; operational definitions, observation windows, and action thresholds should be prespecified and prospectively validated for each clinical use case.

## Beyond thresholds: the patient should be his or her own reference

Conventional medicine is organized largely around thresholds. Blood pressure exceeds a target, heart rate enters an abnormal range, oxygen saturation falls below a cut-off, or weight increases by a predefined amount. Thresholds are useful, but they are an incomplete framework for continuous monitoring.

A systolic pressure of 105 mmHg may be well tolerated by one older person and represent clinically important hypotension in another. A resting heart rate of 90 beats/min may be habitual in one patient but represent a substantial departure from baseline in another. A reduction from 5,000 to 2,500 daily steps can carry information that neither value conveys in isolation.

Frailty makes this distinction particularly important. Frailty represents reduced physiological reserve and increased vulnerability to stressors rather than simply the accumulation of diagnoses (2, 3). A modest physiological perturbation may therefore have very different consequences in a robust and a frail individual.

Continuous monitoring creates the possibility of establishing an individual physiological baseline and evaluating subsequent measurements relative to that baseline. Rather than asking only, “Is this value abnormal?”, wearable systems should increasingly ask, “Has this patient changed, by how much, across which domains, for how long, and in what context?”

This is distinct from using wearables simply to create a digital equivalent of conventional frailty assessment. Recent studies and commentaries have highlighted the potential of daily-life mobility data to reflect functional reserve and health trajectories (4–7). The greater opportunity is to use longitudinal signals to detect change in reserve, potentially before an acute event becomes clinically obvious.

## Multimodal concordance matters more than sensor abundance

A second shift is required from individual sensors toward *multimodal concordance*.

Wearable platforms increasingly generate numerous variables, but more variables do not necessarily produce more information. A small change in resting heart rate may be meaningless. A decline in activity may reflect weather or behavior. Sleep disruption may be transient. Weight can fluctuate because of measurement error. Yet rising heart rate combined with lower activity, fragmented sleep, increasing weight, lower nocturnal oxygen saturation, and new dyspnea tells a different story.

Meaning therefore emerges from patterns. In this framework, magnitude refers to the size of change from an individual's reference state; persistence to the duration or recurrence of that change over a prespecified observation window; multimodal concordance to agreement in timing and direction across clinically relevant signals; and signal quality to the technical reliability and completeness of the data required for interpretation. These elements are not proposed as universal thresholds: their definitions, observation windows, weighting, and action cutoffs should be prespecified and validated for each clinical use case, device configuration, and target population.

Artificial intelligence may help identify these patterns, particularly when changes remain below conventional thresholds. However, algorithmic complexity should not substitute for clinical interpretability. The relevant question is not whether a model can generate a risk score, but whether the underlying pattern can support a proportionate clinical response.

Blood pressure illustrates the challenge. Cuffless measurement is attractive because repeated or continuous observations could reveal variability, nocturnal patterns, medication effects, and hypotensive episodes that conventional measurements miss. Yet calibration, inter-individual variation, vascular properties, body position, movement, and generalizability remain major limitations (8–10). Our own work evaluating photoplethysmography-based blood pressure estimation has similarly demonstrated the importance of intra- and inter-individual variability and the limitations of apparently accurate models when transferred across individuals (11, 12). Accordingly, wearable or cuffless BP trends should be regarded as supportive monitoring information unless the specific device and use case have been clinically validated, and treatment changes prompted by such trends should be confirmed with a clinically validated BP measurement approach.

The solution is therefore not to treat every wearable output as an autonomous biomarker. Signal quality, within-person change, agreement among modalities, and clinical context should jointly determine how much confidence is assigned to a deviation (Figure 1 and Table 1).

**Table 1:** Examples of trajectory-based interpretation and accountable action.

Scenario	Trajectory rather than isolated threshold	Context that modifies interpretation	Confirmatory step	Potential response
HF deterioration	Activity ↓ + HR ↑ + weight ↑ ± sleep/SpO₂ deterioration	Renal function, frailty, diuretic regimen	Symptoms, BP, weight, clinical review	Nurse triage → medication review/teleconsultation
Excess BP lowering	BP trending ↓ + activity ↓ + dizziness/falls	Polypharmacy, autonomic dysfunction, frailty	Clinically validated BP measurement; seated/standing measurements when appropriate	Review BP-lowering therapy only after confirmation and clinical assessment
Intercurrent illness	HR ↑ + activity ↓ + sleep disruption + temperature trend ± SpO₂ ↓	Baseline frailty, lung disease, cognition	Symptoms, temperature/SpO₂ confirmation	Clinical assessment according to severity
Rhythm alert	Persistent rhythm irregularity/PPG abnormality	Ectopy, tremor, perfusion, anticoagulation/fall risk	Wearable or conventional ECG	Diagnose first; treat according to confirmed rhythm

## Older adults are not generic digital-health users

Age should not simply be another covariate in a wearable algorithm.

Older adults may have tremor, edema, altered skin properties, peripheral vascular disease, arrhythmias, gait aids, visual or hearing impairment, arthritis, cognitive impairment, and difficulty maintaining charging or synchronization routines. These characteristics can alter both sensing and engagement. Studies of technology adoption consistently show that older adults may value reassurance, independence, self-awareness, and communication with caregivers while simultaneously encountering discomfort, usability problems, uncertain benefit, privacy concerns, and lack of confidence (13, 14).

More importantly, the same wearable signal can have different clinical meaning depending on geriatric context. Emerging cardiogeriatric literature similarly emphasizes that wearable-derived cardiovascular biomarkers should be interpreted in relation to physiological reserve and frailty rather than as isolated measurements (15).

We therefore propose that wearable interpretation include a contextual layer incorporating, where relevant, frailty, cognition, medication exposure, renal function, recent treatment changes, functional capacity, caregiver availability, and goals of care. These factors should not merely determine whether the patient can operate a device; they should modify how the information is interpreted and acted upon.

Recent work in heart failure has proposed tailoring remote monitoring according to frailty status, emphasizing that robust, prefrail, frail, and palliative populations require different monitoring objectives and workflows (16). Our proposal is complementary but broader: geriatric characteristics should modify the interpretation of physiological trajectories themselves, across diseases and sensor modalities.

## An alert is a request for attention, not a diagnosis

The weakest link in many remote-monitoring systems is not the sensor but what happens after the sensor detects something.

A wearable alert should therefore be considered a request for attention, not a diagnosis. Before implementation, every clinically relevant alert should answer four questions:

## Who receives it? how quickly should it be reviewed? what confirms or refutes it? what happens next?

Stable patterns may require no action. Small transient deviations may justify observation or repeat measurement. Persistent concordant changes may prompt nurse or clinician review. Rapid deterioration or dangerous values may require urgent escalation.

For example, after heart-failure hospitalization, declining activity accompanied by increasing heart rate, weight, and symptoms might trigger nurse review and confirmation of congestion before medication adjustment. Falling blood pressure accompanied by dizziness and reduced activity after intensification of antihypertensive therapy could prompt confirmatory measurement with a clinically validated BP approach and review for overtreatment; treatment should not be changed solely on the basis of an unconfirmed wearable or cuffless BP trend. An irregular-rhythm notification should usually lead to confirmatory ECG rather than immediate therapeutic action.

Heart-failure studies illustrate why this distinction matters. Remote monitoring appears most useful when physiological information is embedded within structured programs incorporating clinical review, education, treatment adjustment, and escalation rather than provided as an isolated stream of measurements (17–19).

A wearable system without a defined response pathway is therefore not yet a clinical intervention. It is a measurement system.

## Evidence should extend from accuracy to net clinical benefit

Wearables are frequently evaluated through analytical performance: correlation, bias, sensitivity, specificity, or classification accuracy. These measures are necessary but insufficient. A useful clinical framework should ask sequentially:

**Analytical validity:** does the device reliably measure the intended signal?
**Physiological validity:** does the signal represent relevant biological change?
**Clinical validity:** does that change identify a clinically meaningful state or trajectory?
**Clinical utility:** does acting on it improve decisions or outcomes?
**Implementation value:** can this be achieved sustainably, equitably, and without disproportionate workload or harm?

Regulatory guidance already emphasizes fit-for-purpose technologies and data quality in remote acquisition (20). For clinical implementation, additional metrics are needed. Studies should report false-alert burden, proportion of alerts resulting in meaningful action, time from signal to review, number needed to evaluate, clinician workload, adherence, patient and caregiver burden, and downstream testing. Ultimately, the appropriate outcome is net clinical benefit, not the volume of data collected.

The equity implications are equally important. Wearable-enabled care could extend specialist oversight beyond hospitals, support community health teams, and enable earlier intervention in geographically dispersed populations. Conversely, smartphone dependence, device costs, digital literacy requirements, proprietary algorithms, and fragmented infrastructure could preferentially benefit healthier and wealthier users. The populations in whom continuous monitoring may be most valuable must therefore be included in validation rather than excluded because they are technically more difficult to study.

## Conclusion

Wearable monitoring in older adults may benefit from moving from continuous measurement toward trajectory-based, context-aware, and accountable care. The clinically relevant signal may often be not an isolated value or population threshold, but a persistent within-person change interpreted across multiple physiological domains and in the context of frailty, cognition, treatment, and functional reserve. The proposed framework—individual baseline, within-person deviation, multimodal concordance, geriatric contextualization, and accountable action—is a conceptual, non-validated model intended to generate testable implementation strategies rather than an established care pathway. Prospective studies are needed to define use-case-specific operational criteria and to evaluate safety, clinical utility, implementation burden, equity, and effects on patient-centered outcomes.

If prospectively validated, the value of wearable medicine in older adults should be judged not by the volume of data collected or alerts generated, but by whether meaningful deterioration can be recognized earlier, unnecessary interventions can be avoided, treatment can be made safer, and independence can be better preserved. The goal is not simply to monitor older adults more intensely, but to determine whether continuous signals can support better, proportionate, and accountable care.

## Data Availability

The original contributions presented in the study are included in the article/Supplementary Material, further inquiries can be directed to the corresponding author.
